# Characterization of Molecular Evolution in Multi-Drug Resistant of *Mycobacterium tuberculosis* by *rpoB* Gene in Patient with Active Pulmonary Tuberculosis from Iranian Isolates

**Published:** 2009-12

**Authors:** Zaker Bostanabad Saeed, Rahimi Mohammad Karim, Adimi Parvaneh, Tayebee Zahra, Masoumi Mozhgan, Pourazar Shahin, Jabbarzadeh Esmail, Shekarabi Mehdi, Pourmand Azarmidokht, Sourkova Larisa Konstantina, Titov Leonid Petrovich

**Affiliations:** 1 *Department of Microbiology and Biology, Islamic Azad University, Parand Branch, Tehran, Iran;*; 2 *Department of Medical Microbiology Department, Islamic Azad University, Medical Branch, Tehran, Iran;*; 3 *Department of Clinical Microbiology, Masoud Laboratory, Tehran, Iran;*; 4 *Department of Clinical Microbiology, Belarusian Research for Microbiology and Epidemiology, Minsk, Belarus;*; 5 *Department of Mycobacteriology, Pasteur Institute of Iran, Iran;*; 6 *Department of Immunology, Faculty of Medicine, Iran University of Medical Science, Iran*

**Keywords:** analysis evolution, *rpoB* gene, *M. tuberculosis*, Iranian isolates

## Abstract

This is the first genetic biodiversity study of Mycobacterium tuberculosis in Iran. Thus, we investigated the genetic patterns of strains isolated in the first survey of anti-tuberculosis drug-resistance by *rpoB* gene as part of the Global Project of Anti-tuberculosis Drug Resistance Surveillance (IAU, Iran). A 411-bp fragment of the *rpoB* gene, containing the sequence of the 81-bp *rpoB* fragment, was amplified by PCR and the *rpoB* gene fragments of tuberculosis strains were sequenced using the Amersham auto sequencer. For analysing tree evolution used method UPGMA and Neighbour-Joining. Clinical isolates (34/163) were analyzed by using sequencing gene *rpoB* and genotyped by program MEGA. The results were compared with the international database. Multi-drug resistant (MDR) was 14% in never treated patients and 8% in previously treated patients. Mutations in *rpoB* gene and *katG* genes were detected in 95% and 84% of the MDR strains, respectively. Two clusters were found to be identical by the four different analysis methods, presumably representing cases of recent transmission of MDR tuberculosis. The other strains are divided into 2 groups: group A – similar to the standard and Eastern strains (China, Taiwan) and group B – strains of another genotype. They are grouped separately on the dendrogram and became prevalent in Iran (they are called Iranian residential strains). This study gives a first overview of the *M. tuberculosis* strains circulating in Iran during the first survey of anti-tuberculosis drug-resistance. It may aid in the creation of a national database that will be a valuable support for further studies.

## INTRODUCTION

*Mycobacterium tuberculosis* has caused tuberculosis (TB) in humans for thousands of years (1, 2), and the World Health Organization (WHO) estimates that one third of the global population is infected with *M. tuberculosis* (3); however, the bacterium has remained an enigma. The global resurgence of TB highlights the need for an improved understanding of its epidemiology and its evolutionary biologic features. Recent advances in molecular characterization of *M. tuberculosis* isolates, which index variation in insertion sequences (4) and repetitive genomic elements (5, 6); have elucidated clusters of identical and closely related strain families (7–9). These findings have provided insights into regional (10) and national (11) epidemiologic features. However, these techniques may be less suited to global population and evolutionary analyses, and integrating information obtained from different approaches is complex (12). Genomic comparisons have identified genetic variation for population screening; however, these analyses are limited to those sites that vary between the compared genomes and are potentially misleading (13–15). Nucleotide sequences provide robust, portable, and comparable data for studying population variation. The mutational processes that generate this variation are understood, and sequence data have been successfully used in the study of bacterial epidemiology, population biology, and evolution (16). The complete genome sequences (15–18) provide access to all regions of the chromosome and facilitate such studies. However, high-throughput gene sequencing of structural genes (19) and host immune system protein targets (20) in *M. tuberculosis* isolates indicated low levels of sequence diversity.

In *M. tuberculosis*, resistance to antibiotics occurs because of genomic mutations in certain genes, such as the *katG* gene for isoniazid (INH) resistance and the *rpoB* gene for rifampicine resistance (RIF) (4). In contrast to several other pathogens with MDR phenotypes, plasmid or transposon-mediated mechanisms of resistance have not been reported in *M. tuberculosis* (13–15). Since resistance to antimicrobial drug in *M. tuberculosis* is exclusively due to genomic mutations, the bacterium would benefit from an increased mutation rate.

Although extensive genomic sequencing was performed in both studies, comparable sequence data were obtained on a limited number of highly selected isolates.

Ongoing research is focused on identifying the factors responsible for the worldwide spread of the W-Beijing strains and their ability to adapt and enhance their pathogenisity or virulence. Identifying a possible mechanism for increased adaptation of these bacteria to the human immunologic host defense system or human interventions such as anti-TB treatment is of the utmost importance. Such mechanisms may indicate how the bacterium adapts to the host, a prerequisite for an enhanced accumulation of genomic mutations associated with resistance.

Despite an unusually high degree of relatedness at the genetic level, the five species comprising the *M. tuberculosis* complex differ in their host range, their phenotypes and ability to cause disease in humans. Variations in virulence are even observed, in animal models of the disease, between members of the *M. tuberculosis* species. Comparative genomics has been applied to the *M. tuberculosis* complex in an attempt to understand the molecular basis for these differences.

The genome sequences of the paradigm strain, H37Rv, and a recent clinical isolate, CDC1551, comprise 4.41 and 4.40 Mb, respectively, and harbour ∼4,000 genes coding for proteins. In contrast, the genome of *M. bovis* strain AF2122/97 at 4.34 Mb is significantly smaller as the result of a series of deletion events that have removed ∼80 genes.

The molecular characterization of the isolates collected during this study was not carried out and there is no information on the major circulating clades of *M. tuberculosis*. The mutations involved in drug resistance and the question of whether the Beijing family of the *M. tuberculosis* complex had spread into Iran have not been studied until now. Molecular epidemiological studies have showed the widespread of this family and its association with drug-resistance (6–8).

In recent years, the genetic typing of *M. tuberculosis* complex (MTC) strains has been widely used to support conventional epidemiological investigations of TB outbreaks and as a tool for studying transmission dynamics.

In this study, we investigated the genetic patterns of strains isolated in the first survey of anti-tuberculosis drug-resistance to rifampicin (RIF) and isoniazid (INH) by *rpoB* and *katG* genes as part of the Global Project of Anti-tuberculosis Drug Resistance Surveillance.

## MATERIALS AND METHODS

### Mycobacterial strains and Drug susceptibility testing

From total 463 strains, 34 isolates were recovered from cultured sputum samples resistance to rifampicine and 28 isoniazid-resistant were isolated from sputum of patients with active pulmonary tuberculosis, from March to December 2005. All strains were cultured on Lowenstein-Jensen solid medium and identified to the species level using TCH (2-thiophene carboxylic acid) and PN99B (paranitrobenzoic acid) selective media or by standard biochemical procedures (5).

Drug Susceptibility testing were performed by BACTEC system and CDC procedure (isoniazid 1 μg/mL, rifampicine 40 μg/mL, streptomycin 10 μg/mL, or ethambutol 2 μg/mL) (5), using absolute concentration method on slants with H37Rv strain of *M. tuberculosis* as positive control. Resistance was defined as growth on solid media containing graded concentrations of drugs with radiological data and PPD skin tests.

### PCR Amplification

DNA extraction was purified using Fermentas kit’s (K512). A 411-bp fragment of the *rpoB* gene and 209-bp fragment of *katG* was amplified by PCR with primers *rpoB-F* (5-TACGGTCGGCGAGCTGATCC-3) and *rpoB-R* (5-TACGGCGTTTCGATGAACC-3) and *katG-F* 5-GAAACAGCGGCGCTGATCGT-3, *katG-R* 5-GTTGTCCCATTTCGTCGGGG-3(22). PCR was carried out in 50 μL of a reaction mixture containing 50 mM KCl, 10 mM Tris (pH 8.0), 1.5 mM MgCl2, 5 μM of deoxynucleoside triphosphates (dNTPs), 1U *Taq* polymerase, 20 pmoles of each set of primers, and 6 μM of chromosomal DNA (24). For *rpoB* fragment Samples were then subjected to one cycle at 94°C for 5 min, followed by 36 cycles at 94°C for 1 min, 57°C for 1 min, 72°C for 1 min, and a final cycle at 72°C for 10 min to complete the elongation of the PCR intermediate products. For *katG* the following thermocycler parameters were used: initial denaturation at 94°C for 5 min; 42 cycles of denaturation at 94°C for 1 min; primer annealing at 57°C for 1 min; extension at 72°C for 1 min; and a final extension at 72°C for 10 min. PCR products were then run on 2% agarose gels and examined for the presence of the 411-bp and 209 bp band after ethidium bromide staining. The DNA Extraction were performed on agaros using Sigma Kit (124K6083) and the products were checked and purified on the gel electrophoresis and purified *rpoB* and *katG* segment were amplified. The resultant DNA amplifications were used for sequencing.

### DNA sequencing of the *rpoB* fragment

Sequencing of 411-bp fragment were done using the same forward and reveres primers; 33 cycles of denaturation at 94°C for 30min; primer annealing at 54°C for 30 sec; extension at 72°C for 90 sec, A 411-bp fragment of the *rpoB* gene extracted from tuberculosis strains were sequenced by Amersham auto sequencer and Amersham Pharmacia DYEnamic ET Terminator Cycle Sequencing Premix Kits. Alignment of the DNA fragments (*rpoB*) was carried out with the help of MEGA software (Gen bank_PUBMED/BLAST).

### DNA sequencing of the *katG* fragment

Sequencing of 209-bp fragment of *katG* gene was amplified by PCR using forward and reveres primers; 33 cycles of denaturation at 94°C for 30 sec; primer annealing at 48°C for 45 sec; extension at 60°C for 4 min. The *katG* gene fragments of tuberculosis isolates were sequenced using the Amersham auto sequencer and Amersham Pharmacia DYEnamic ET Terminator Cycle Sequencing Premix Kits.

### Data analyzing of DNA sequencing

DNA sequences from *rpoB* and *katG* fragment were analyzed by “Blast” program (http://www.ncbi.nlm.nih.gov/BLAST/). In this manner, sequences of standard strains of H37RV, CDC1551 were used as control and compared with test strains. Comparisons of all sequences, mutations were performed, by applying “Mega” and “DNA MAN” program. Alignment of the DNA fragments (*rpoB*) was carried out with the help of MEGA software (Gen bank_PUBMED/BLAST). The obtained data were analyzed and edited with DNAMAN programs.

### Mutations spectrum and frequency analysis

DNAMAN is a sequence analysis software package for IBM compatible computers with Microsoft Windows 95/98 or NT/2000 systems. This package provides effective and convenient tools for molecular biologists to deal with frequently used analyses in research. It contains text editor for fragments sequence alignment. The text editor is identical to WINDOWS WordPad. The editor allows deleting or pasting any text or sequence to files.

### Cluster analysis by Neighbor-Joining method

The basic unit of data used in Neighbor-Joining (Nearest Neighbor) cluster analysis is the similarity coefficient derived from any one of the similarity analysis formula. A similarity matrix derived from sequential pairwise comparisons of two most similar operable taxonomic units (OTU) or clusters of OTUs is used to construct a phenogram. This dendrogram is setup with the distance matrix using the Neighbor-Joining method (Saitou and Nei, 1987, Mol. Biol. Ecol. 4:406–425). Phylogenetic tree shows related homologies between any two sequences in a multiple alignment.

### Unweighted pair-group method using arithmetic averages

Unweighted pair-group method using arithmetic averages (UPGMA) is one of the most frequently used cluster analysis methods an is widely applied in the construction of dendrograms based on electrophoretic fingerprint patterns, hybridization matrices, and nucleic acid sequences. With UPGMA, an OTU, or every OTU in a cluster of OTUs is joined to a known cluster on the basis of the average (mean) distance between all combinations of pairs of OTUs. Thus, UPGMA calculates the average distance from one cluster of OTUs to a comparison cluster, where each OTU in the cluster is given equal weight. This dendrogram is setup with the distance matrix using the UPGMA method (Sneath and Sokal, 1973, Numerical Taxonomy, San Francisco, USA). The matrix can be built up only with Observed Divergence method. Dendrogram shows related homologies between two sequences or groups.

## RESULTS AND DISCUSSION

### Study of population and Bacterial strains

A total of 62 patients with sputum smear-positive pulmonary TB was included in the survey to determine the initial prevalence and acquired resistance to the principal anti-TB drugs. Of these, 41 (66%) had never been treated for tuberculosis and the other 21 (34%) had previously received treatment. Of the 463 isolates, 378 (81%) were sensitive to all four evaluated drugs and 62 (19%) showed resistance to rifampicine, (34) with other drugs or isoniazid, and (28) with other drugs.

Total resistance to INH and RIF in never treated patients was 100% (28 patients) and 100% (34 patients), respectively. Most of the patients with drug-resistant isolates (16/2–24%) were aged between 15 and 54 years.

### Bacterial strains

All samples were cultured and identified as *M. tuberculosis* by biochemical methods. 34 Rifampicine-resistance and 28 isoniazid-resistance *M. tuberculosis* clinical isolates (including MDR strains) were subjected.

### Drug susceptibility

All 34 isolates of rifampicine resistant examined were resistant to rifampicine, isoniazid (80%), streptomycin (90%) and 18 isolates (48%) were resistant to etambutol. In this study we found two strains Mono-resistance to rifampicine. All 28 isolates isoniazid resistant examined were resistant to isoniazid, rifampicine (65%) streptomycin (82%) and 8 isolates (28%) were resistant to etambutol. Mono-resistance to isoniazid was observed for 4 isolates (14%) in this study.

### PCR Amplification and DNA sequencing analysis for *rpoB*

**Figure 1:**
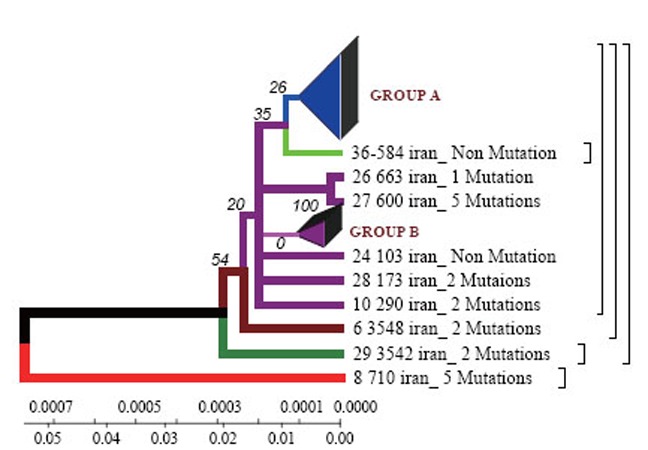
Phylogenetic dendrograms of *M. tuberculosis* strains isolated from Iranian patients within the time period of 70 years by Neighbour-Joining method. The phylogenetic tree, generated using the Neighbour-Joining method with 1000 bootstrap replicates and distance calculated using the number of different sSNP loci (http://www.megasoftware.net/).

60 mutations and 13 micro deletions were identified in 29 rifampicine-resistances MBT (85%). In 5 rifampicine-resistances MBT isolates (15%) no mutations were found in the core region of the *rpoB* gene. Of 60 found mutations 6 silent (8.3%) and 54 (91.7%) were missense. Most of detected deletions were identified in codon 510 GAG/_AG (12.5%). All silent mutations were localized in codon 507, missense mutations revealed 23 types of amino acid substitutions. Most frequent mutated codons in Iranian strains were 523 GGG/GG_, GGG/GCG and 526 CAC/TAC, CAC/CGC, CAC/AAC, CAC/TTC, CAC/CAA, CAC/_GC (six types of mutations, Table 1 and Table 2). Mutations in codons 510, 507, 531 were observed in 27%, 24%, and 21% of isolates and correspondingly Mutations in codon 523 resulted in Gly523Ala replacement and in codon 531 Ser_531_Leu and Ser_531_Phe. We observed 6 alleles in codon 526, 3 alleles in triplets 507, 508, 513. In 6 strains (18%) harboured single mutations placed in codons 526, 510, while isolates with multiple mutations revealed double 34%, triple 22% and quadruple 3% of the strains. 12% of strains harboured 5 mutations (Table 1 and Table 2) (Fig. 1).

**Table 1:** Frequency of amino acid and nucleotide changes of different codons in *rpoB* gene of rifampicin-resistant strains of *M. tuberculosis* isolated in Iran that has been registered in Genbank, EF628339-EF628368

Codon and Amino acid change	Nucleotide change	Frequency	Isolates

531 Ser→Leu	TCG→TTG	5 (6.78%)	3708, 441, 163 (2), 29 (2), 710
531 Ser→Phe	TCG→TTC	2 (2.78%)	159, 163
526 His→Tyr	CAC→TAC	4 (5.5%)	3062, 108, 36, 159
526 His→Asn	CAC→AAC	1 (1.39%)	167
526 His→deletion	CAC→_GC	1 (1.39%)	165
526 His→Arg	CAC→CGC	3 (4.2%)	663, 600, 710
526 His→Phe	CAC→TTC	2 (2.78%)	36 asli, 161
526 His→Gln	CAC→CAA	1 (1.39%)	163
510 Gln→deletion	CAG→_AG	9 (12.51%)	90, 633, 411, 73, 23, 3708, 441, 163 (2), 29 (2), 3542
507 Gly→Ser	GGC→AGT	1 (1.39%)	3542
507 Gly→Gly	GGC→GGT	6 (8.3%)	19, 10, 33, 10 (2), 163, 710
507 Gly→Asp	GGC→GAT	1 (1.39%)	159
508 Thr→Ala	ACC→GCC	1 (1.39%)	290
508 Thr→Pro	ACC→CCC	3 (4.2%)	3548, 3542, 663
508 Thr→His	ACC→CAC	2 (2.78%)	710, 163
509 Cys→Asp	AGC→GAC	1 (1.39%)	600
511 Leu→Ser	CTG→CCG	2 (2.78%)	303–281, 165
511 Leu→Val	CTG→GTG	1 (1.39%)	600
512 Ser→Tyr	AGC→GGC	2 (2.78%)	36 asli, 710
512 Ser→Gly	AGC→GCC	1 (1.39%)	159
513 Gln→Asn	CAA→AAT	1 (1.39%)	36 asli
513 Gln→Stop	CAA→TAA	1 (1.39%)	159
513 Gln→Glu	CAA→GAA	1 (1.39%)	600
516 Asp→His	GAC→CAC	1 (1.39%)	663
519 Asn→Lys	AAC→AAG	1 (1.39%)	600
520 Leu→deletion	CCG→C_G	1 (1.39%)	303–281
523 Gly→Ala	GGG→GCG	16 (22.24%)	167, 161, 290, 3548, 173, 23, 19, 10, 33, 10 (2), 3708, 441, 163 (2), 303–281, 165, 710
523 Gly→deletion	GGG→GG_	1 (1.39%)	29 (2)
527 Lys→deletion	AAG→deletion	1 (1.39%)	36 asli

**Table 2:** Data for *rpoB* mutations (single, double, triple, quartile and five) in rifampicin-resistant *M. tuberculosis* strains isolated from Iran

Frequency of Mutation	Number of codon	Number of Isolates	Isolate number

Non mutation			23 (2)−28−584, 103, 29
1 Mutation	526	3	
	510	3	3062, 108, 36, 90, 633, 411
2 Mutation	523–526	2	167, 161
	508–523	2	290, 3548
	510–523	2	173, 23
	507–508	1	3542
	507–523	4	19, 10, 33, 10 (2)
3 Mutation	510−523−531	4	3708, 441, 163 (2), 29 (2)
	508−516−526	1	2
	511−520−523	1	663
	511−523−526	1	303-281, 165
4 Mutation	507−508−526−531	1	163
5 Mutation	512−513−526−527−531	1	36 asli
	507−508−512−523−526	1	710
	507−512−513−526−531	1	159
	509−511−513−519−526	1	600

### PCR Amplification and DNA sequencing analysing for *katG*

**Figure 2:**
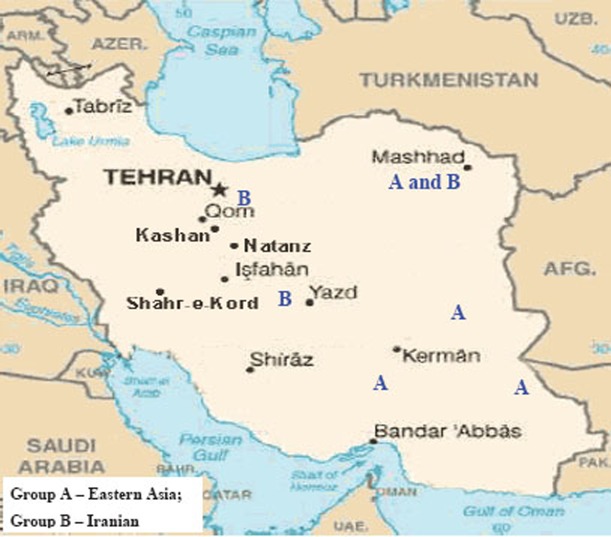
Distribution of genetic groups of *M. tuberculosis* on the territory of Iran.

All 28 samples were cultured and identified as *M. tuberculosis* by PCR method that reveals 49 mutations in all stains. From 26 isoniazid-resistance 2 strains showed no mutation and in 21 strains mutations were observed in codon 315, revealing three types of mutations consist of AGC→ACC (Ser→Thr) (80%), AGC→AGG (Ser→Arg) (5%) and AGC→AAC (Ser→Asn) (15%). One type of mutation obtained in codon 299 indicating GGC→AGC and changes in amino acid Gly→Ser. In codon 311(18%) (*katG*) only one base change were obtained Asp→Tyr (GAC→TAC) in nine strains demonstrate a nonsense mutation (Table 3). Furthermore only one mutation observed in codons 311, 299 and 322. And in 12 strains one mutation in codon 315 (42.8%), 7 strains 2 (25%), 5 strains 3 (17.8%) and 2 isolates 4 mutations (7.1%) were obtained respectively (Table 3) (Fig. 2).

**Table 3:** Frequency of amino acid and nucleotide changes of different codons in *katG* gene of rifampicin-resistant strains of *M. tuberculosis* isolated in Iran that has been registered in Genbank, EU884576-EF884601

Codon	Frequency		Amino acid change	Nucleotide change	Isolates

**1 Mutation**					
315	12	10	Ser→Thr	AGC→ACC	3542, 600, 90, 10, 19, 441, 28, 3708, 29, 3548
		1	Ser→Arg	AGC→AGG	98 (1383)
		1	Ser→Asn	AGC→AAC	1619 (MAC)
**2 Mutation**					
309	2	2	Gly→Val	GGT→GTT	633, 12
315	2		Ser→Asn	AGC→AAC	
315	1	1	Ser→Thr	Asn→Ile	633
322			Asn→Ile	AAC→ATC	
315	2	2	Ser→Thr	Pro→Ala	
324			Pro→Ala	CCG→GCG	108, 303 (281)
299	1	1	Gly→Ser	GGC→AGC	411
311			Tyr→Tyr	GAC→TAC	
311	1	1	Tyr→Tyr	GAC→TAC	610
315			Ser→Thr	AGC→ACC	
**3 Mutation**					
299	1	1	Gly→Ser	GGC→AGC	159 (MDR)
311			Tyr→Tyr	GAC→TAC	
315			Ser→Thr	AGC→ACC	
299	3	3	Gly→Ser	GGC→AGC	163, 167
311			Tyr→Tyr	GAC→TAC	165 (MDR)
322			Asn→Ile	AAC→ATC	
299	1	3	Gly→Ser	GGC→AGC	161 (MDR)
311			Tyr→Tyr	GAC→TAC	
324			Pro→Ala	CCG→GCG	
**4 Mutation**					
299	2	2	Gly→Ser	GGC→AGC	290 and 710 (MDR)
311			Tyr→Tyr	GAC→TAC	
315			Ser→Thr	AGC→ACC	
322			Asn→Ile	AAC→ATC	

### Evolutionary analysis of relationships of *M. tuberculosis* strains prevalent on the territory of Iran

Sequencing of 81 bp fragment of *rpoB* gene was carried out for 29 strains. From the of sequences obtained, a dendrograms was constructed by UPGMA and Neighbour-Joining methods in DNAMAN software.

*Homologic dendrogram* was constructed by UPGMA method using distance matrix. According to the dendrogram, the Iranian strain 710 is closely related to the strains from Taiwan, China and India (BLAST software). The other strains are supposed to have originated from this one.

*Phylogenetic dendrogram.* With the help of Neighbour-Joining method the data obtained confirm the results of Neighbour-Joining method for the Iranian strain 710 (Fig. 1).

According to the dendrogram one strain from the town of Zabol is older than the rest of the strains (it is about 77 years old), and it was considerably changed after its appearance in Iran and was resistant to anti-tuberculosis drugs (Fig. 4). This strain contains five mutations. When it appeared in Iran it possessed drug resistance. The other strains similar to each other are located on the same branch of the dendrogram (Fig. 1).

The other strains are divided into 2 groups: group A – similar to the standard and Eastern strains (China, Taiwan), they are about 10 years old. These strains were changed in Iran and acquired mutations (Fig. 4); group B – strains of another genotype. They are grouped separately on the dendrogram and became prevalent in Iran (they are called Iranian residential strains) (Fig. 2).

**Figure 3:**
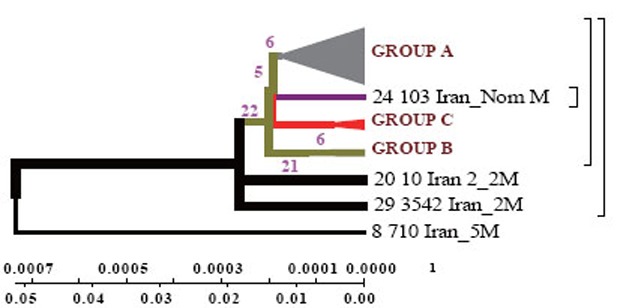
Dendrogram constructed by Minimum method for Iranian strains. The phylogenetic tree, generated using the minimum method with 1000 bootstrap replicates and distance calculated using the number of different sSNP loci (http://www.megasoftware.net/).

DNAMAN software allowed to obtain general classification of *M. tuberculosis* collected from the patients from different regions of Iran. In the dendrogram constructed by Minimum method all strains are divided into three genetic groups and in each of them there are closely related strains (Fig. 3).

Strains of group A are similar to the standard ones, and formed not long ago; group B comprises two strains of earlier origin than the others, and it’s obvious that they were changed after their appearance in Iran; group C comprises four strains which appeared not long ago.

**Figure 4:**
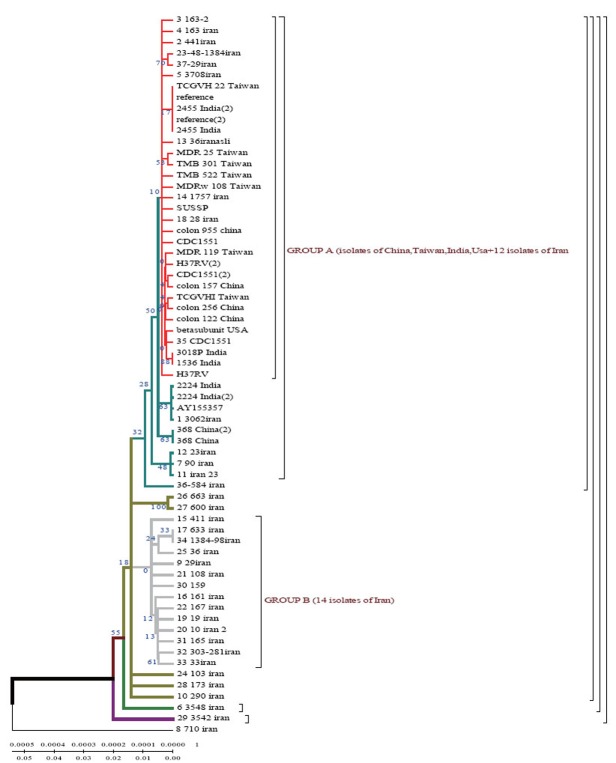
Evolutionary dendrogram of *M. tuberculosis* strains constructed by UPGMA method.

According to the dendrogram constructed by UPGMA method all strains are divided into two genetic groups. Some of the strains differ in genotype and divergence time and are located separately (Fig. 4). 60% of Iranian strains diverged as a result of mutations from the same *M. tuberculosis* strain 710 similar to the strains from China, Taiwan and India. 20% of the strains had the highest similarity with the ones from Taiwan. One isolate (from Zabol region) 710 is related evolutionary to ancestral *M. tuberculosis* family. In this sample there are no mutations in codon 531. Strain 163 located separately and the number of strains combined in one group originated from it. Four isolates belong to the standard *M. tuberculosis* strains (H37RV, CDC1551), and on of these four is similar to Indian strain 3062 Iran (Fig. 4).

**Figure 5:**
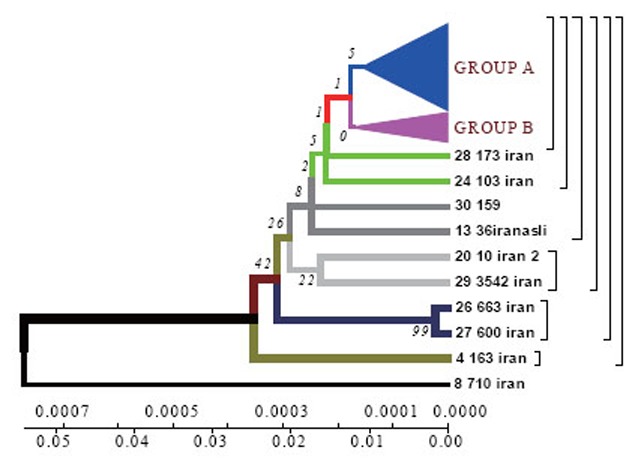
Dendrograms and their classification obtained by UPGMA method. The phylogenetic tree, generated using the UPGMA method with 1000 bootstrap replicates and distance calculated using the number of different sSNP loci (http://www.megasoftware.net/).

23 isolates are combined in groups and have Iranian origin. Five isolates diverges from the primary sample: one from Tehran, one from Kurdestan, three ones are similar to the strains from Taiwan (TMB 522). All the strains from Iranian patients are divided into two genetic groups: A – similar to Eastern strains (Taiwan, China, India, Japan, and Korea), they are about 11 years old; B – Iranian residential strains which appeared more than 20 years ago (Fig. 5).

In the cause of study of mutations in *rpoB* gene in the strains from Iranian patients the most frequent changes were found in codons 523 and 526. In the isolates from the patients with primary tuberculosis (12 strains) the changes were detected in codons 526 (28.5%), 523 (21.4%), 510 (14.2%). In the isolates from the patients with secondary tuberculosis (22 strains) mutations were found in codons 526 (13.5%), 523 (23.7%) and 510 (11.8%).

In Iranian *M. tuberculosis* strains in *katG* gene the most affected codons were 315 and 311. In the strains from the patients with primary tuberculosis the changes were detected in codons 315 (25%), 311 (0.9%), 299 (0.9%). In the strains from the patients with secondary tuberculosis mutations were found in codons 315 (36.8%), 299 (18.4%), 311 (21%). According to the dendrograms constructed for *rpoB* gene the strain 710 from the town of Zabol is more closely related to the Chinese strains. The other strains are supposed to have diverged from this strain. Iranian strain 710 has the greatest similarity with the strains from China, Taiwan and India. Strain 710 from Iran has the highest similarity of its genome with the strains from China, Taiwan and India. It appeared about 77 years ago.

Using Neighbour-Joining method evolutionary dendrograms were obtained for Iranian strains which were divided into two genetic groups: group A – strains more related to Eastern ones (Taiwan, China, India, Japan, and Korea); group B – Iranian residential strains.

Evolutionary dendrograms constructed for the first time for *M. tuberculosis* strains collected in Iran show that strain 710 of *rpoB* gene collected from the town of Zabol is closely related to the strains from China, Taiwan and India. The other strains are supposed to have diverged from this one. All strains analysed collected from Iranian patients are divided into 2 genetic groups: group A – similar to Eastern strains (Taiwan, China, India, Japan, Korea) (they are about 11 years old); group B – Iranian residential strains which appeared about 23 years ago.

Construction of evolutionary dendrograms using sequences of *M. tuberculosis* resistance genes collected in Iran and bioinformatics analysis are recommended to study epidemic process of tuberculosis.
